# The Impact of a Music- and Movement-Based Intervention on Motor Competence, Social Engagement, and Behavior in Children with Autism Spectrum Disorder

**DOI:** 10.3390/children12010087

**Published:** 2025-01-14

**Authors:** Chayma Kanzari, Aymen Hawani, Karim Ben Ayed, Maher Mrayeh, Santo Marsigliante, Antonella Muscella

**Affiliations:** 1 High Institute of Sport and Physical Education of Kef, University of Jendouba, El Kef 7100, Tunisia; kanzarichayma95@gmail.com (C.K.); ben.ayedk1@yahoo.fr (K.B.A.); 2 Higher Institute of Sport and Physical Education (Ksar Saïd), University of Manouba, Manouba 2010, Tunisia; aymen.hawani@issep.uma.tn (A.H.); meher.mrayeh@issep.uma.tn (M.M.); 3 Physical Activity, Sport and Health, Research Unit (UR18JS01), National Observatory of Sport, Tunis 1003, Tunisia; 4 Department of Biological and Environmental Science and Technologies (DiSTeBA), University of Salento, 73100 Lecce, Italy; santo.marsigliante@unisalento.it

**Keywords:** autism spectrum disorder (ASD), music, movement, physical activity, intervention, motor functions, stereotypical behavior, social engagement

## Abstract

Background/Objectives: The main objective of this manuscript is to evaluate the effects of training, music, and movement intervention on motor functions, social engagement, and behaviors in autistic children. Methods: Twenty-one children with a diagnosis of mild autism spectrum disorder (ASD), with an age range of 5-to-13 years, were divided into two groups: the experimental group (*n* = 10) and the control group (*n* = 11). All participants were examined before (T0) and after the intervention (T1) to evaluate their motor functions (Bruininks–Oseretsky Motor Performance Test (BOT-2)), maladaptive behavior (RCS (Response to Challenge Scale)), and enjoyment and engagement (PACES (Physical Activity Enjoyment Scale)). Results: Statistical analysis showed that music and movement intervention significantly improved motor functions such as balance and bilateral coordination (*p* < 0.0001), social engagement (*p* = 0.002), and adaptive behaviors (*p* = 0.005) in children with ASD. Our research supports the feasibility of music and movement intervention and documents the interest in participating in children with ASD. Conclusions: This study demonstrates the benefits of movement and music interventions and can be considered a useful way to manage autism spectrum disorders in the future.

## 1. Introduction

Autism spectrum disorder (ASD) is a neurodevelopmental condition characterized by persistent deficits in communication and social interaction, and repetitive behaviors [1].

Its diagnosis is based on two axes: on one hand on the presence of persistent deficits in language, communication, and socialization, and on the other hand on the presence of a stereotypical or repetitive nature of movement [2,3]. Nowadays, physical activity is considered an important determinant of health status for children and adolescents to grow up healthy [4], so it is important that they are physically active. Children diagnosed with ASD tend to have a higher rate of motor impairment [5] and movement disorders such as poor coordination [6], balance [7], gross walking skills, postural stability, and movement [8]. These deficiencies make physical activity, in some cases, a real challenge. Given the prevalence of motor deficits in children with ASD, it is essential to use reliable tools to assess their motor competence and monitor potential improvements following interventions.

Motor competence refers to an individual’s ability to execute a wide range of motor tasks with proficiency, encompassing both fine and gross motor skills. It is a critical construct in child development, as it influences physical, cognitive, and social outcomes [9,10]. In the context of this study, motor competence was assessed using the Bruininks–Oseretsky Motor Performance Test, Second Edition (BOT-2), a validated and widely used instrument designed to evaluate various dimensions of motor skills, including balance, coordination, strength, and agility [11]. The BOT-2 provides a comprehensive framework to measure motor abilities in children and has been extensively utilized in research involving children with developmental disorders, including ASD [10,12,13].

For this reason, parents and teachers recognize the importance of children’s movement during physical activity, as it contributes to their personal development and reflects the joy of life.

While numerous studies have explored the benefits of physical activity interventions for children with ASD, fewer have emphasized the broader significance of physical activity and the risks associated with inactivity for children with disabilities in general. For instance, early interventions focusing on motor skills have been shown to improve balance, coordination, and posture stability [14,15,16,17]. Gabriels et al. [18] demonstrated that therapeutic horse-riding programs significantly enhance self-regulation, socialization, and communication in children with ASD. Similarly, Najafabadi et al. [19] found that their program (Sports, Play and Active Recreation for Kids (SPARK)) improved both motor skills and social interactions among participants. Pan et al. [20] highlighted the impact of table tennis training, showing significant improvements in motor competence and executive function. Additionally, aquatic physical activity has been identified as a valid intervention for ameliorating motor and social skills [21,22,23,24].

The enjoyment of play as well as its importance in developing motor, social, and communication skills has prompted professionals to develop a variety of play-based interventions to promote these skills in children with ASD. However, the modalities of interventions and designs used to evaluate their effectiveness vary greatly [25].

Behavioral benefits have also been noted following physical activity and music interventions. For example, Ren et al. [26] highlighted that dance and movement activities, often combined with music, can reduce maladaptive behaviors and promote emotional regulation in children with ASD. Similarly, Ye et al. [27] demonstrated that music-based interventions can reduce aggressive behaviors and improve emotional responses in children and adolescents.

These findings underscore the potential of physical activity to enhance physical abilities, promote social engagement, and reduce stereotypic behaviors.

Despite these promising results, limited research exists on interventions that combine structured movement with music, which may offer additional benefits by engaging multiple cognitive and emotional domains simultaneously.

According to these findings, regarding the type of physical activity interventions for the treatment of children with ASD and according to literature researchers who have taken different approaches based on their personal views, there is disagreement over which is the most effective intervention. This survey was designed to find out which type of physical training could be beneficial for children with ASD.

This study aimed to address gaps in the literature on the combined effects of music and movement interventions by assessing their impact on motor functions, social engagement, and behaviors in children with ASD. It was hypothesized that participation in physical activity and sports would positively influence self-perception and self-esteem in children with ASD.

Furthermore, it was predicted that children who participate in an 8-week program of sports and leisure activities will show greater improvements compared to children in the control group who are not part of the intervention. Specifically, the following was hypothesized: (1) the intervention group would show significant improvements in motor competence, including balance, coordination, and strength, as measured by the BOT-2; (2) the intervention group would show increased social engagement, reflected by increased interaction and participation during group activities; (3) the intervention group would experience reductions in maladaptive behaviors and improvements in emotional and cognitive self-regulation; and (4) the intervention group would report higher levels of enjoyment and motivation for physical activity than the control group.

## 2. Materials and Methods

### 2.1. Participants

A preliminary calculation using G*Power 3.1.9.4 software (version 3.1, University of Düsseldorf, Düsseldorf, Germany) showed that a sample size of 20 participants would be required to detect a mean effect size of d = 1.01 with 95% power and an alpha level of 0.05. The primary statistical test chosen for this study was the paired samples *t*-test.

Twenty-one children (aged 5–13 years) with ASD were enrolled at the Jendouba Autism Centre (13 boys and 8 girls). Children with a diagnosis of autism spectrum disorder were enrolled based on DSM-V-TR criteria [28].

After consultation with a therapist and a speech therapist responsible for all individuals with disabilities, children with a diagnosis of mild ASD who did not have severe behavioral problems, who had the ability and willingness to participate in music activities and movement classes, and who were free of a severe physical disability, medical problem, or diagnosis that would preclude participation in the intervention were included in this study.

Therefore, children with chronic diseases, verbal communication problems, physical disabilities, or acute injuries at the time of screening were excluded. The inclusion criteria for this study were as follows: (1) a diagnosis of autism spectrum disorder (ASD) corresponding to Level 1 or Level 2 support needs based on DSM-5-TR criteria; (2) no severe behavioral issues that could interfere with participation; (3) ability and willingness to participate in music and movement activities; and (4) absence of severe physical disabilities, medical conditions, or other diagnoses that would preclude participation.

Participants were randomly assigned to the experimental or control group using specialized software to ensure an unbiased and balanced distribution of baseline characteristics between the groups. The randomization process was conducted to ensure that both groups were comparable in terms of age, gender, and severity of ASD diagnosis. To further reduce potential bias, researchers involved in data collection were blinded to participants’ group assignments during pre- and post-intervention assessments. This method aimed to support the internal validity of this study and reduce systematic errors in group assignment. The characteristics of the children are presented in Table 1.

**Figure 1:**
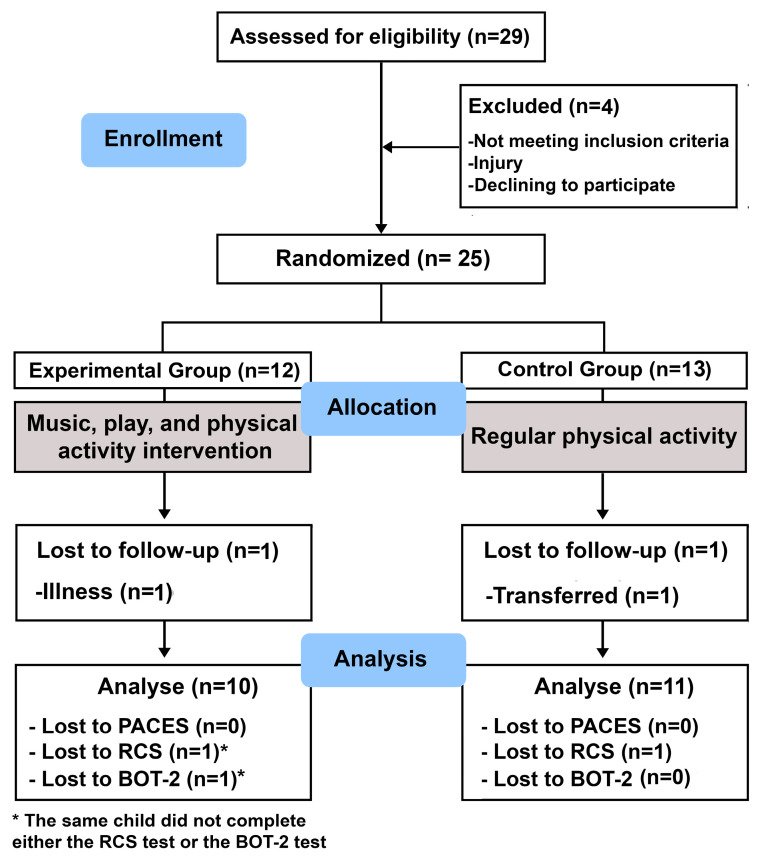
Flow diagram. Experimental design: PACES, Physical Activity Enjoyment Scale; RCS, Response to Challenge Scale; BOT-2, Bruininks–Oseretsky Motor Performance Test.

Additionally, children were excluded from data analysis if they did not complete the program or did not complete the questionnaire due to illness, transfer, or inability to complete it. The randomized controlled trial design showed in Figure 1 was used. For the analysis, we employed “blind” researchers who performed the measurements and statistical data analysis.

The experimental group (*n* = 10) included 5 females and 5 males with ASD. The protocol was explained to the parents and an informed consent form was signed for their children to participate in this project. The structured physical activity program was used as the intervention for the 10 children in the experimental group. The other 11 children with ASD (control group) participated in their regular physical activity program at the autism center. Their program lacked specific intervention elements (e.g., no music, play, or engaging and creative components), but included activities such as walking, running, or basic exercises.

### 2.2. Ethical Considerations

The tenets of the 2013 Declaration of Helsinki were adhered to in the experimental protocol. Ethical approval (approval reference: CPP N 13/2024, 11 April 2024) was granted by the local research ethics committee of the Higher Institute of Sports and Physical Education of Kef. Parents were informed of the protocol and signed a consent form for their children’s participation in this project, ensuring that all parties were fully informed of this study’s purpose, procedures, potential risks, and benefits. In order to maintain their autonomy, parents or participants were assured of their right to withdraw from this study at any time without negative consequences. Confidentiality was maintained through the anonymization of the teams, the use of group names, and the exclusion of sensitive information. In view of the young age of the participants, special precautions were taken to ensure their comfort and understanding of the requirements of this study, such as the provision of a safe environment and the minimization of potential physical or emotional harm.

### 2.3. Procedure

This study was conducted from August to October. It lasted 10 weeks in total, consisting of 1 week of pre-testing (T0), 8 weeks of specific training (movement and music intervention), and 1 week of post-testing (T1). Participants in the control group (*n* = 11) were exposed to the same care in the children’s center for people with ASD as the intervention group (occupational therapist, speech therapist), but they did not participate in the intervention exercise sessions: they participated in their regular physical activity.

Participants were assessed before and after the 2-month intervention. Children’s enjoyment and engagement was assessed using the Physical Activity Enjoyment Scale (PACES); secondly, children also used the Response to Challenge Scale (RCS); finally, the Bruininks–Oseretsky Motor Performance Test (BOT-2) was used to assess motor function such as balance and coordination. All tests were administered on 3 consecutive days, 48 h apart.

### 2.4. Intervention Program

In the present study, the intervention protocol combined music, physical activity, and play-based motor activity. Each of these activities individually has been shown to have positive effects on children with ASD [29,30,31].

**Figure 2:**
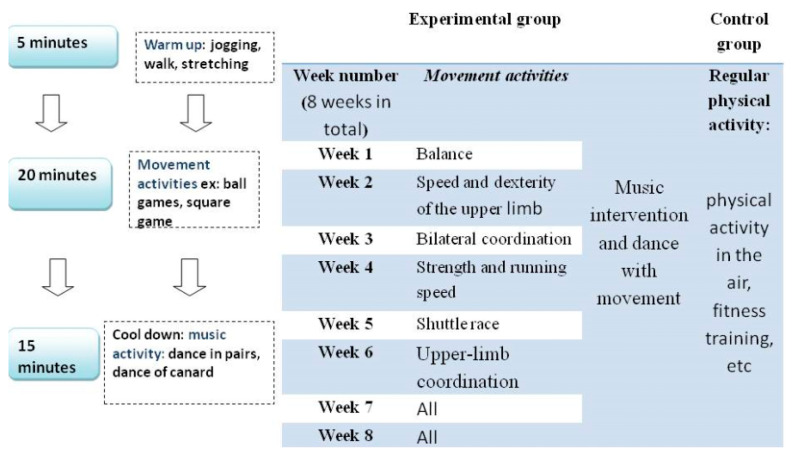
Explanatory figure of the intervention protocol. Physical activity in the air refers to movements where the feet leave the ground, including jumps, leaps, rope skipping, trampolining, or rhythmic gymnastics.

An intervention with an experimental group was used to investigate the effects of 8 weeks of physical activity, including movement and musical activities, on motor skills, social interactions, and qualitative characteristics. Twenty-four training sessions of 45 min were carried out over eight weeks, 3 times a week. This program consisted of three parts: (1) warm-up; (2) movement activities; and (3) musical activities. Each session included 5 min of warm-up, followed by 3 min of recovery after warm-up, 20 min of movement activities, and, finally, 15 min of musical activities. The training protocols of the experimental group are detailed in Figure 2.

The control group did not receive any intervention. They were exposed to the same care as the intervention group in the children’s center for people with ASD (occupational therapist, speech therapist) and were invited to continue their daily programs (physical activity in the air, fitness training, etc.).

### 2.5. Measures

All participants underwent a comprehensive assessment at baseline, before the start of the 2-month intervention, and immediately after its completion. The purpose of this assessment was to evaluate the participants’ baseline conditions and to measure any changes or improvements resulting from the intervention.

#### 2.5.1. Assessment of Social Engagement and Children’s Enjoyment

The modified Physical Activity Enjoyment Scale (PACES) is an effective measure of children’s enjoyment of physical activity and can be used for children with intellectual disabilities [32,33] and with ASD [29,34].

It consists of 18 bipolar statements on a 7-point Likert scale (e.g., “I like it”–“I hate it”), with scores summed to provide an overall enjoyment score. The statements were made understandable for the participants’ reading level. This tool offers a comprehensive view of children’s attitudes toward physical activity, influencing their engagement and motivation. A caregiver helps account for the child’s emotional states or engagement levels that may not be readily expressed verbally.

#### 2.5.2. Assessment of Stereotypical Behavior

The Response to Challenge Scale (RCS) was developed to measure children’s cognitive, affective, and motor regulation abilities in an exercise context [12]. Children were asked to complete a series of tasks of varying complexity, ranging from relatively simple activities, such as jogging in a field, to more challenging tasks, such as jumping over a student. Performance on these tasks is assessed across three dimensions of self-regulation: (a) motor control, which is the child’s ability to effectively coordinate their physical movements while performing the tasks; (b) affective control, which is the child’s ability to manage their emotions, particularly frustration or excitement that may arise during challenging tasks; and (c) cognitive control, which is the child’s ability to think strategically and make decisions while performing the motor tasks.

#### 2.5.3. Assessment of Motor Functions

The Bruininks–Oseretsky Motor Performance Test (BOT-2) is designed to assess motor skills such as balance and coordination in individuals aged 4-to-21 years [11,12]. The BOT-2 is composed of eight distinct subscales that assess a range of motor skills, including both fine and gross motor function. These subscales are as follows:

(a) Fine Motor Control: Assesses precision and coordination in tasks requiring detailed hand movements.
(b) Manual Coordination: Assesses the ability to perform tasks requiring hand–eye coordination and dexterity.
(c) Body Coordination: Measures balance and the ability to control body movements in dynamic tasks.
(d) Strength: Assesses muscle strength through a variety of physical activities.
(e) Agility: Assesses the ability to change direction quickly and maintain control during movement.
(f) Overall Motor Performance: Provides a comprehensive overview of the child’s motor skills.
(g) Bilateral Coordination: Assesses the ability to use both sides of the body together in a coordinated manner.
(h) Speed and Endurance: Measures how quickly and efficiently tasks can be completed over time.

The BOT-2 contains 53 specific items, involving a variety of activities such as cutting a circle, copying a square, bouncing a ball, and standing on one leg. Each item is designed to reflect real-life motor challenges, allowing assessors to collect meaningful data about a child’s motor skills.

### 2.6. Statistical Analysis

Data analysis was performed using SPSS version 20 for Windows (SPSS Inc., Chicago, IL, USA). Values are presented as averages and standard deviations. The normality of the data sets was verified using the Kolmogorov–Smirnov test. The equality of error variances was tested using the Levene test. The univariate two-factor analysis of variance [Group (Experimental vs. Control) × measurement (T0 and T1)] was used to determine the differences between the experimental conditions.

A post hoc Bonferroni test was applied following the two-way repeated-measures ANOVA to examine pairwise differences between groups (experimental vs. control) and time points (T0 vs. T1). This test was chosen to control the Type I error in multiple comparisons and ensure result validity. Standardized effect size analysis (Cohen’s d) was used to interpret the magnitude of the differences between the variables. The meaning statistic was set at *p* < 0.05.

## 3. Results

### 3.1. Motor Functions

Table 2 shows the results of the Bruininks–Oseretsky Motor Performance Test (BOT-2) measured before and after 8 weeks of the intervention. Participants completed the post-intervention Bruininks–Oseretsky Motor Performance Test (BOT-2) (Table 2). After 8 weeks of intervention, the experimental group had a very high retest score (*p* < 0.0001). In addition, this group improved significantly before and after the intervention.

A repeated-measures ANOVA was conducted to assess the effects of the intervention on manual dexterity, bilateral coordination, balance, and speed. The analysis revealed a significant group effect for manual dexterity (F(1, 19) = 5.610; *p* = 0.034; d = 0.554) and bilateral coordination (F(1, 19) = 3.196; *p* = 0.010; d = 0.738) in the experimental group. Additionally, the experimental group showed significantly higher scores for balance and speed compared to the control group, with significant improvements observed from pre-intervention (T0) to post-intervention (T1) (*p* < 0.0001; d = 3.351). These results suggest that the intervention had a marked positive effect on physical performance, particularly in the experimental group.

### 3.2. Maladaptive Behaviors

Table 3 shows the values of motor/physical control, affective/emotional behavior, and cognitive control of the experimental group during their sessions before and after 8 weeks.

Following the unadjusted two-factor analysis of variance, statistical analysis revealed a group effect (*p* = 0.044). However, there was no effect of time (*p* = 0.325) and no interaction between time and group (*p* = 0.38). After 8 weeks of intervention, scores in the experimental group improved significantly (*p* = 0.005; d = 1.572) compared to the control group.

### 3.3. Children Enjoyment

**Figure 3:**
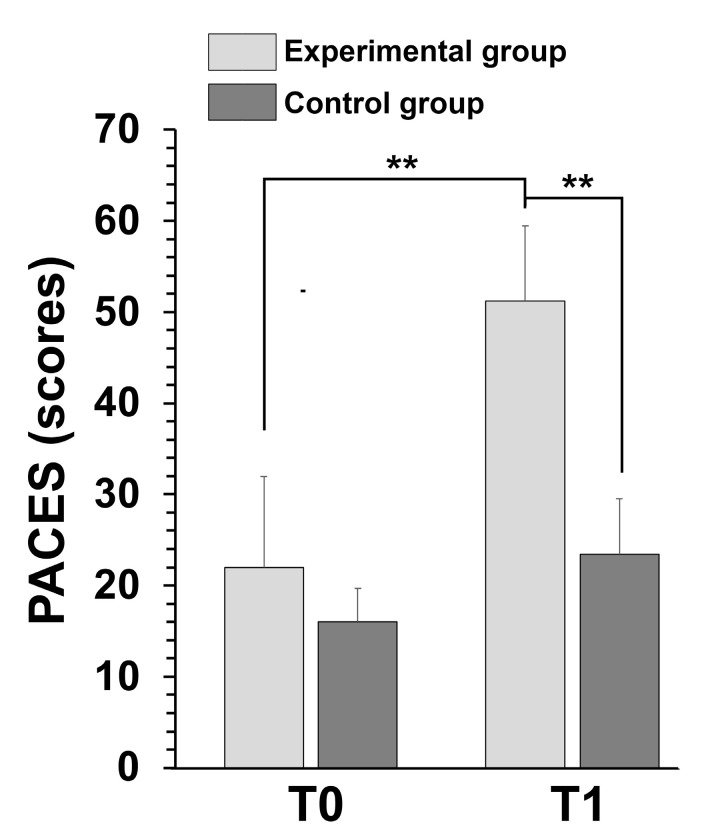
The mean scores of the Physical Activity Enjoyment Scale (PACES) before and after the training program in both groups. ** *p* < 0.01 by ANOVA.

PA enjoyment was assessed using the PACES. Statistical analysis revealed a greater group effect (*p* = 0.004) and time effect (*p* = 0.002) in the experimental group compared to the control group. A significant interaction between group and time was observed (*p* = 0.005), with raw scores improving in both experimental conditions. After 8 weeks of intervention and rehabilitation, PACES scores were very high in the experimental group compared to the control group (*p* = 0.001). Similarly, scores improved significantly in the experimental group post-intervention (*p* = 0.002) (Figure 3).

## 4. Discussion

The purpose of this study was to evaluate the effects of an 8-week intervention combining physical activity, music, and play on the motor skills, social behaviors, and emotional well-being of children with ASD. This integrated approach aimed to enhance the motor, social, and emotional development of children with ASD through engaging and multisensory activities.

A significant amount of scientific evidence has shown that physical activity benefits children with ASD, as it improves cognitive functions, academic performance, and motor skills [20,35,36,37,38].

Physical activity interventions, which typically last 8–14 weeks, include a variety of activities such as swimming, running, yoga, horseback riding, and exergaming [29,39]. As a therapeutic tool, play also plays a fundamental role in the development of children with ASD, as it contributes to improving emotional regulation, their social skills, and their motor and cognitive skills. Motor play, which includes structured and spontaneous movements, can particularly improve motor skills in children with ASD, especially regarding motor coordination, dexterity, and balance [40]. In addition, structured and facilitated play can improve social skills and communication in children with ASD, promoting active participation and cooperation [40,41,42]. It is known that play offers a natural opportunity for learning, as it stimulates engagement and promotes the acquisition of skills in an engaging and motivating way in all children.

The professional use of music as an intervention strategy in healthcare, educational, and everyday contexts, to improve the quality of life and enhance the physical and spiritual well-being of individuals, developed as a systematic intervention in the twentieth century [43].

Some studies showed that music therapy, which uses musical experiences to improve language, social, and communication skills, promotes social interaction, reduces challenging behaviors, and stimulates enthusiasm and participation [44,45]. In children with ASD, musical activities have been shown to improve social behavior and promote brain connectivity, brain plasticity, and language development [46]. The combination of music and movement has been shown to improve motor coordination, synchronization, and communication skills while creating a structured framework for social interactions [47,48]. Music and movement combined eliminated repetitive behaviors in children with ASD and improved social skills and creativity [26]. Similarly, dance and movement therapies have been demonstrated to reduce stereotyped behaviors and improve emotional regulation, motor control, and social functioning in individuals with developmental disabilities, including ASD [49].

The integration of play with motor activities and music therapy, in particular, can potentially optimize therapeutic effects, providing a multisensory experience that facilitates the improvement of motor and social functions [50,51]. The combination of physical activity and music has been poorly explored as an opportunity to improve the coordination, emotional self-regulation, and social interactions of children with ASD. The results are still only partially complete and need further confirmation.

In this context, an intervention was developed that combined physical activity, music, and games to integrate the different positive aspects and offer a stimulating and engaging environment capable of promoting motor, social, and emotional development in children with ASD.

By integrating movement, play, and music into a classroom setting, this study sought to improve motor skills, promote social interactions, and improve overall behavioral functioning in children with ASD.

According to the first hypothesis, the results suggest that an 8-week intervention combining physical, musical, and play activities would lead to improvements in motor skills, including manual dexterity, balance, bilateral coordination, and speed, in children with ASD. The results support this hypothesis, demonstrating significant increases in these motor areas for the experimental group. These positive results are consistent with previous studies that explored movement- and physical activity-based interventions [29,31] and are consistent with studies demonstrating the positive effects of music therapy on motor development [52,53].

The second hypothesis posited that the intervention would also result in improvements in social behaviors and emotional regulation. The findings support this hypothesis, as we observed significant improvements in maladaptive behaviors, emotional control, and social interactions within the experimental group. This is in line with prior studies that suggested that physical activity and play can help reduce maladaptive behaviors and enhance social functioning in children with ASD [30].

Our study also sought to investigate whether the combination of music and movement would have a synergistic effect on motor and social functions. The integrated approach of movement and music, as suggested by Lakes et al. [29], proved beneficial in improving motor control, manual dexterity, and bilateral coordination, which are essential for daily activities and nonverbal communication in children with ASD.

This supports findings from Sanglakh Goochan et al. [53], who demonstrated that music-synchronized motor activities improve motor skills in children with ASD.

However, there is one study that does not report statistically significant effects on coordination in people with ASD after a physical activity intervention [54]. These conflicting findings in the literature regarding the effects of physical activity and movement interventions on children with ASD mean that future studies should investigate potential moderating factors, such as the intensity and frequency of the interventions or individual differences in participants.

The improvement in motor skills for the experimental group compared to the control group in our study can probably be explained by the combination of movements and music.

The results also revealed a significant improvement in maladaptive behaviors in the experimental group, indicating that the intervention not only improved motor skills but also had a positive impact on the children’s emotional and behavioral well-being. This outcome is consistent with previous research that highlighted the effectiveness of play and physical activity in reducing maladaptive behaviors in children with ASD [30].

The increase in pleasure associated with physical activity was particularly significant in the experimental group. The higher scores on the Pleasure Assessment Scale at the end of the intervention, compared to the control group, suggest that the combination of physical and musical activity not only improves motor functions but also increases motivation and, consequently, children’s engagement. These results agree with the results of other studies that have highlighted the importance of pleasure and motivation as key factors for the success of interventions in children with ASD [29,31]. Thus, the intervention based on play and music helped to create a positive and stimulating environment, which increases children’s participation and facilitates the development of motor and social skills. On the other hand, it is known that play, as a therapeutic tool, not only promotes social interaction but also encourages children’s motivation to participate actively, improving the effectiveness of the intervention [30,41].

The results of this study align with the growing amount of evidence in the literature that highlight the effectiveness of interventions that combine physical activity and sensory stimuli, such as music, in improving motor functions, behavior, and engagement in children with ASD. The combination of movement and music is effective in promoting the development of fine and gross motor skills, reducing maladaptive behaviors, and increasing motivation to participate, as highlighted by previous studies [29,31].

Furthermore, the literature suggests that interventions involving play, such as the one described in this study, are particularly effective in promoting social integration and improving communication skills in children with ASD [30,55]. The results obtained in this study, particularly the finding that pleasure and active participation significantly increased in the experimental group, confirm that play-based approaches are particularly suitable for promoting the well-being of children with autism spectrum disorders. In fact, the play also stimulates intrinsic motivation and promotes more active and satisfying participation, elements that are crucial for the success of therapeutic interventions.

Although this study demonstrates that the intervention is highly effective, it is necessary to recognize its methodological limitations and the potential influence of confounding factors. First, the small sample size limits the generalizability of the results. Furthermore, this study did not account for external factors, such as previous exposure to physical activity, that could have influenced the results. Additionally, a key limitation of the current study is its short duration and lack of long-term follow-up, which limits our ability to assess the sustainability of the observed effects on motor function, social engagement, and behaviors in children with ASD. Future research should implement longer-term interventions and incorporate follow-up assessments to determine whether the improvements observed in this study are maintained over time.

Future studies addressing these issues could provide further insights into the mechanisms driving these improvements and their practical applications in different contexts.

## 5. Conclusions

In summary, the results of this study support the validity of an intervention combining physical activity, music, and play as a way of improving motor skills, social behaviors, and motivation in children with ASD. Therefore, such an intervention may be a valuable tool for professionals and coaches to support the development of motor functions and social integration of children with ASD.

## Figures and Tables

**Table 1:** Children’s characteristics.

Groups	Age (yr)	Height (cm)	Body Mass (kg)	Physical Education Experience (yr)	Autism Level
**Experimental**	7.8 ± 1.94	116.0 ± 4.2	25.7 ± 0.06	0.9 ± 0.06	1.5 ± 0.58
**Control**	8.4 ± 3.03	117.1 ± 3.3	25.8 ± 0.03	1.1 ± 0.03	1.2 ± 0.42

Data are mean ± SD; Autism Level, according to the Diagnostic and Statistical Manual of Mental Disorders (DSM-5) [28].

**Table 2:** Average motor functions scores before and after the interventions.

	Experimental Group		Control Group	
	T0	T1	T0	T1
**Manual Dexterity**	4.3 ± 3.4	6.8 ± 1.7 †§	1.7 ± 0.9	2.2 ± 1.0
**Bilateral Coordination**	0.2 ± 0.4	1.8 ± 1.3 †§	0.6 ± 1.0	0.6 ± 1.1
**Balance**	1.2 ± 0.8	3.3 ± 0.5 †§	0.7 ± 1.1	0.4 ± 1.0
**Speed**	0.2 ± 0.4	3.7 ± 2.0 †§	0.3 ± 0.7	0.5 ± 1.1
**Coordination of the Lower Limb**	0.5 ± 1.2	3.7 ± 2.0 †§	1.2 ± 2.6	1.5 ± 2.6
**Strength**	0.5 ± 0.8	2.8 ± 0.8 †	0.7 ± 1.2	1.5 ± 1.9
**Total**	6.8 ± 4.6	21.8 ± 4.5 †§	5.2 ± 5.1	6.7 ± 5.8

Data are expressed as mean ± S.D. †, significant difference between pre (T0)- and post (T1)-intervention. §, significant difference between the experimental and control group after the intervention (T1). The statistical significance was set at *p* < 0.05.

**Table 3:** Scores for stereotypical behaviors pre (T0)- and post (T1)-program training in both groups.

	Experimental Group		Control Group	
	T0	T1	T0	T1
**Motors/Physical control**	2.3 ± 2.1	6.3 ± 2.5	1.6 ± 2.8	2.5 ± 3.5
**Affective or Emotional Behaviors**	19.0 ± 13.1	31.3 ± 9.3	21.8 ± 9.9	21.8 ± 9.6
Cognitive Control	13.5 ± 9.8	24.0 ± 7.7	12.0 ± 13.1	10.9 ± 10.2
Total	33.8 ± 23.3	61.7 ± 18.1	35.4 ± 23.3	35.2 ± 22.0

Data are expressed as mean ± S.D.

## Data Availability

The raw data supporting the conclusions of this article will be made available by the authors on request. The data is not publicly available for privacy, legal or ethical reasons.
